# Immune infiltration in aggressive papillary craniopharyngioma: High infiltration but low action

**DOI:** 10.3389/fimmu.2022.995655

**Published:** 2022-11-01

**Authors:** Yanfei Jia, Lin Ma, Kefan Cai, Bochao Zhang, Wentao Wu, Youchao Xiao, Ning Qiao, Siming Ru, Lei Cao, Hua Gao, Songbai Gui

**Affiliations:** ^1^ Department of Neurosurgery, Beijing Tiantan Hospital, Capital Medical University, Beijing, China; ^2^ Department of Neurosurgery, Lanzhou University Second Hospital, Lanzhou University, Lanzhou, China

**Keywords:** papillary craniopharyngioma, hypothalamic invasion, Immune infiltration, interleukin-1α, interleukin-6

## Abstract

Papillary craniopharyngiomas (PCPs) are biologically benign but clinically aggressive lesions hence affect the quality of life. The expression of inflammatory mediators and regulation of the immune microenvironment in PCPs have not been investigated much. In this study, for the first time, we assessed the immune cell infiltration and immune cell signatures in PCPs by analyzing the bulk-RNA sequencing data and immunohistochemical staining. Additionally, we performed qRT-PCR analysis to detect inflammatory mediators interleukin-1α (*IL1A*) and interleukin-6 (*IL6*) in different aggressive groups and then developed the *IL1A* and *IL6* prediction models for defining the degree of hypothalamic invasion. Lastly, we defined differentially expressed genes related to invasiveness and implemented enrichment analysis to them. Our results indicated that PCPs are in a state of high immune infiltration but low action with abundant inflammatory cells. High infiltration of neutrophils may lead a low active immune microenvironment. Furthermore, the high expression level of *IL1A* and *IL6* was positively correlated with the invasion of PCP tumors in the hypothalamus. These findings provide new pathological insights into the underlying mechanism of the immune microenvironment in PCP tumors. Moreover, *IL1A* and *IL6* might serve as potential therapeutic targets for PCP tumors, especially to prevent their invasion into the hypothalamus.

## Introduction

Craniopharyngiomas are benign slow-growing tumors of the sella region derived from the remnants of Rathke’s pouch. Globally, they constitute 1.2–4.6% of all intracranial tumors and account for 0.5–2.5 new cases per 1 million population per year (1, 2). The two histological subtypes of craniopharyngioma, adamantinomatous craniopharyngioma (ACP) and papillary craniopharyngioma (PCP), differ in their development and histologic features. ACP occurs in both children and adults, whereas PCP occurs almost exclusively in adults (3). And PCPs frequently harbor somatic BARF^V600E^ mutations that result in the activation of the mitogen-activated protein kinase signaling pathways (4).

Traditional craniotomy surgery or endoscopic endonasal surgery is the mainstay of treatment for patients with craniopharyngioma. Nonetheless, gross total excision remains a challenge due to frequent infiltration and adhesions to surrounding tissues (5). In some patients with craniopharyngiomas, the tumor has been observed to erode and destroy the hypothalamus, in which case resection could lead to postoperative hypothalamic damage, resulting in severe obesity, neuroendocrine deficiencies, and poor quality of life (6, 7). Meanwhile, because of adhesion and invasion into the hypothalamus, tumor recurrence was observed in up to 36% of cases treated with gross total resection, and this rate increased up to 67% in those treated with subtotal resection (8). Therefore, solving the problem of tumor adhesion or invasion into the hypothalamus would not only have important theoretical and realistic significance but would also be essential for achieving a good clinical effect in the treatment of craniopharyngiomas.

**Figure 1:**
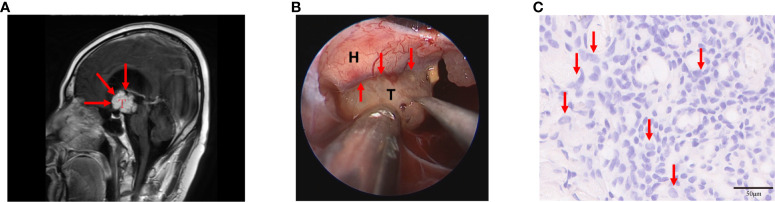
Puget grade 2 papillary craniopharyngioma (PCP) tumor with magnetic resonance imaging (MRI) image, surgical findings, and histopathological results. **(A)** This tumor caused the dislocation, compression, and destruction of the hypothalamus as shown in the MRI image (indicated by the red arrow), T: PCP tumor. **(B)** The tumor was surrounded by edema, its brittleness was increased, and a considerable amount of yellow fibrin exuded from the tumor during the operation (indicated by the red arrow), T: PCP tumor, H: hypothalamus. **(C)** Histopathology showed abundant neutrophils infiltrated in tumor tissue (indicated by the red arrow), hematoxylin-eosin staining 20×.

Craniopharyngiomas with hypothalamic adhesion or invasion can be evaluated based on preoperative Puget grading system (9). Grade 0 craniopharyngiomas have no contact with the floor of the third ventricle, whereas grade 1 craniopharyngiomas have contact with or compress the hypothalamus anterior to the mammillary bodies. Grade 2 craniopharyngiomas result in dislocation, compression, or destruction of the hypothalamus, including the mammillary bodies or the area dorsal to them (**Figure 1A**) (6, 10). According to online reports, grade 2 tumors were found to manifest the worst outcome (6, 9, 11). Interestingly, we found that grade 2 PCP tumors mostly have macroscopic characteristics during operation as follows: tumor surrounded by irregular edema, increased tumor brittleness, and considerable yellow fibrin exuded from the tumor, among others (**Figure 1B**). Pathological tissue section exhibited that the tumor and peripheral tissue of the hypothalamus coexist with neutrophil infiltration, which is a general inflammatory outcome (**Figure 1C**). With these discoveries, we may reasonably conclude that inflammation plays a key role in hypothalamic invasion in PCPs, especially Puget grade 2 tumors.

Since A Stevens et al. found that craniopharyngiomas contain considerable numbers of T-helper/inducer cells and B-cells in 1988 (12), more and more evidence showed the correlation between craniopharyngiomas and inflammation. Ming chen et al. demonstrated that patients with craniopharyngiomas had higher levels of pre-operative white blood cell and lymphocyte, and the PCP group had higher neutrophil count and neutrophil lymphocyte ratio in peripheral blood samples than ACP and healthy control groups (13). Previous study suggests the involvement of innate immune response in pathology of craniopharyngioma cyst formation (14). Followed-up researchers confirmed that numerous cytokines, chemokines, and inflammatory mediators are expressed in craniopharyngiomas including IL-1A, IL-1B, IL-6, IL-8, IL-10, IL-18, TNF-α, TNF-β, IFN-γ, MCP-1 and CXCL1 (6, 15–18). Mori et al. demonstrated highly elevated levels of IL-6 in the ACPs cyst fluid and posited that IL-6 plays an important role in the inflammatory reaction associated with ACPs (19). Then Multiple studies have also identified high levels of IL-6 in ACPs, thus made IL-6 became a potential therapy target (15, 16). Moreover, the pattern of cytokine expression is compatible with inflammasome activation, possibly triggered by the cholesterol crystals present in human ACPs (15). Studies reported that high levels of alpha defensin proteins, which are present in neutrophils, are involved in the ACPs mediated by an inflammatory response. Furthermore, inflammatory factor levels were significantly reduced after treatment with intracystic interferon alpha (14, 20). However, the studies of inflammatory mediators and the immune microenvironment in PCPs, especially the relationship between hypothalamic invasion and inflammatory reaction, are currently limited (6, 21). Considering the importance of inflammation in craniopharyngiomas, there is an urgent requirement for further study on immune-related gene expression and regulatory mechanisms in PCP tumors.

In this study, we aimed to describe the immune infiltration in PCP tumors by analyze the bulk-RNA sequencing data gained from 11 human PCP tumor samples. Furthermore, we focused on immune cells that form the major non-tumor constituents and identified specific signatures of the immune cells of grade 0 to grade 2 PCP tumors. Besides, we assessed functional state of immune microenvironment of PCPs and made possible explanation. Additionally, we have detected the expression level of inflammatory mediators interleukin-1α (*IL1A*) and interleukin-6 (*IL6*) in different aggressive groups and developed the *IL1A* and *IL6* prediction models, which was successfully applied to define the degree of invasion to the hypothalamus. Lastly, we defined differentially expressed genes related to the invasiveness of PCPs and implemented enrichment analysis to them.

## Methods

### Patients and samples

This study was approved by the ethics committee of the Beijing Tiantan Hospital of Capital Medical University, and all patients enrolled in this study provided signed informed consent.

We obtained 11 craniopharyngioma tumor samples and five normal brain tissues from the Beijing Tiantan Hospital between May 2019 and June 2020. The tumor samples were obtained after the surgical treatment and diagnosed *via* postoperative pathology results. Furthermore, normal brain tissues from five patients with craniocerebral trauma were used for comparative purposes, and all the tissue samples were conserved in RNAlater^®^ (Thermo Fisher Scientific, Waltham, MA) at -80°C for subsequent RNA sequencing.

Corresponding clinical information, including age, gender, clinical symptoms, hypothalamic invasion, tumor size, location, and prognosis status, were also collected. Finally, these tumor samples were divided into three groups based on the hypothalamic invasion level using the Puget grading system: (1) grade 0 was a low aggressive group, n = 3; (2) grade 1 was a medium aggressive group, n = 4; (3) grade 2 was a high aggressive group, n = 4.

### Total RNA extraction

Total RNA was extracted from PCP tissues using Trizol reagent (Thermo Fisher Scientific, Waltham, MA, USA). The concentration and quality of total RNA were evaluated using a NanoDrop ND 1000 spectrophotometer (NanoDrop Technologies, Wilmington, DE, USA), and the integrity of total RNA was determined using the Agilent 2100 Bioanalyzer (Agilent Technologies, Santa Clara, CA, USA). High-quality RNA samples (RNA integrity number≥7) were used for further analysis.

### RNA sequencing analysis

A total of 16 tissue samples (11 PCP tumor tissues and 5 normal tissues) were subjected to whole transcriptome analysis using high-throughput sequencing. Ribosomal RNA (rRNA) was removed from total RNA (1 μg) using the Ribo-Zero Gold rRNA kit (Illumina, San Diego, CA, USA). Strand-specific cDNA libraries were prepared using the TruSeq RNA Sample Prep Kit (Illumina, San Diego, CA, USA) according to the manufacturer’s instructions without purification and enrichment of mRNAs. Briefly, RNA was ligated with T4 RNA Ligase 1 at the 3’ ends and T4 RNA Ligase 2 at the 5’ adapters, followed by reverse transcription into cDNA. After PCR amplification, the products were purified using polyacrylamide gel electrophoresis.

The resulting libraries were validated using the Agilent Bioanalyzer 2100 system (Agilent Technologies, CA, USA), followed by sequencing using the HiSeq™ 2000 platform (Illumina, San Diego, CA, USA). Subsequently, we applied Cutadapt adapters (version 1.16) to trim the raw reads and used the FastQC software (V 0.11.7) to obtain quality control reports of the sequence reads. Finally, the sequencing data were aligned to the human reference genome (hg38) using the STAR software (V2.3.0), and the count data were normalized as the fragments per kilobase of transcript per million data after filtering read count files with low expression. The original sequence count data was normalized by transcript per million (TPM) and then calculated by log(x+1).

### Immunocyte infiltration analysis

Gene set enrichment analysis (GSEA) is a powerful computational algorithm that determines whether a pre-defined set of genes shows differences between two groups (22). The single-sample GSEA (ssGSEA), an extension of GSEA, was performed using the R “GSVA” package (V1.30.0) to quantify the tumor infiltration of 28 immune cell types based on the TPM values of each sequenced sample (23). To evaluate the relationship between the level of tumor invasiveness and tumor-infiltrating immune, the R package “ESTIMATE” was used to obtain the estimated scores of tumor purity and to evaluate the overall level of immune cell infiltration in low (n=3) and high (n=4) aggressive groups (24). Moreover, to evaluate the synergy effect among immune cells, the correlations between 28 immune cell infiltration types in tumor samples were calculated based on Spearman’s correlation analysis.

### Identification of differentially expressed genes associated with tumor aggressiveness

To compare the immune infiltration degree of different aggressive tumors, analysis of variance (ANOVA) was conducted on immune scores among low (Puget grade 0, n = 3), medium (Puget grade 1, n = 3), and high (Puget grade 2, n = 4) invasive groups. To find the differentially expressed genes (DEGs) between low and high hypothalamic invasion groups, the R package “limma” (V3.29.0)was performed (25). DEGs were selected with the threshold log2 fold change (FC) > log2(1.5) and an adjusted p-value < 0.05. To present an overview of the differentially expressed profiles of the transcripts, volcano plots were generated using the R “ggplot2” package (V3.3.0).

DEGs were identified, and functional enrichment analysis was carried out to explore their functions. Gene Ontology (GO) enrichment analysis and Kyoto Encyclopedia of Genes and Genomes (KEGG) pathway enrichment analysis for DEGs were performed with the R package “clusterprofile” (V4.4.3) (26). Based on the GO categories, the genes were identified with different GO terms according to their respective characteristics: molecular functions (MFs), biological processes (BPs), and cellular components (CCs). Additionally, KEGG pathways were used for pathway enrichment analysis. The false discovery rate was set at 0.05.

### Quantitative reverse transcriptase-polymerase chain reaction

We collected another 36 tumor samples from PCP patients that included the grade 0 group (n=16) and grade 2 group (n=20) and determined their inflammatory factor *IL1A* and *IL6* gene expression by quantitative reverse transcriptase-polymerase chain reaction (qRT-PCR).

Total RNA was isolated from tumor samples using a Trizol reagent kit (Invitrogen Co., Carlsbad, CA, USA). cDNA was synthesized with oligo (deoxythymidine) primers at 50°C, using the SuperScript III FirstStandard Synthesis System for RT-PCR (Invitrogen Co., Carlsbad, CA, USA). Quantitative real-time PCR was run in triplicate using TaqMan PCR Master Mix (Roche Applied Science, Indianapolis, IN, USA), and real-time quantitative primers were designed and purchased from Applied Biosystems (Life Technologies, Carlsbad, CA, USA).

The details about primers used in this study are showed in **Table 1**.

**Table 1:** Primer sequences used in this study.

Gene	Forward Primer 5’-3’	Reverse Primer 5’-3’
*GAPDH*	GAAGGTGAAGGTCGGAGTC	GAAGATGGTGATGGGATTTC
*IL1A*	GTCGGGAGGAGACGACTCTA	GCAACTCCTTCAGCAACACG
*IL6*	GCCTTCTTGGGACTGATGCT	TGCCATTGCACAACTCTTTTCT

The PCR cycling conditions were as follows: initial denaturation and enzyme activation at 95°C for 30 s, followed by 40 cycles of denaturation at 95°C for 5 s, annealing at 60°C for 20 s, and extension at 72°C for 1 min. The copy number of the transcripts was normalized against that of the *GAPDH* transcripts for each sample. All samples were run in triplicate and quantified as the number of cycles (Ct) after which the fluorescence exceeded the background threshold minus the Ct for *GAPDH*. Relative expression levels were calculated using the 2^-ΔΔCT^ method, comparing grade 0 and 2 groups. The identity and purity of the amplified product were checked through the analysis of the melting curve carried out at the end of amplification.

### Immunohistochemistry

Immunohistochemistry was conducted using the Leica BOND-MAX automated system and Bond Polymer Refine Detection Kit (Leica Microsystems, DS9800) according to the manufacturer’s protocol. Briefly, tissue sections were deparaffinized with Bone Dewax Solution and treated with the Epitope Retrieval Solution 1 (Citrate buffer) or Solution 2 (EDTA-buffer pH 8.8) at 98 °C for 20 min. After washing, 3–4% (v/v) hydrogen peroxide was added to block endogenous peroxidase activity. Tissues were washed and incubated with primary antibodies for 30 min followed by polymer for 15 min and developed with 3,3-diaminobenzidine (DAB) for 10 min. For the negative control, the primary antibody was omitted and replaced with blocking buffer containing the same amount of IgG from non-immune rabbit or mouse serum. IHC results were reviewed by pathologists.

### Construction of a diagnostic model for determining the invasive level

The diagnostic model establishment was based on logistic regression analysis: the low (Puget grade 0, n = 16) and high (Puget grade 2, n = 20) aggressive groups were subject to binary variable assignment, with 0 and 1 for the low and high aggressive group, respectively. The selected prognosis-related inflammatory factors were applied in the least absolute shrinkage and selection operator (LASSO) model using the R package “glmnet” (V2.0-10) (27). The scoring formula was established using a linear combination of the regression coefficient, which was acquired from LASSO. The 36 samples were split into two groups, with 70% (n = 26) as the training and 30% (n = 10) as the test datasets.

### Statistical analysis

Statistical analysis was performed using SPSS version 19.0 software (IBM Corp. Chicago, IL, USA). All experiments were performed in triplicates, and data are presented as the mean ± standard deviation. Unpaired Student’s t−test was used to compare differences between two groups, whereas one−way ANOVA, followed by Tukey’s *post hoc* test, was used to compare differences between multiple groups. Values with *p* < 0.05 were considered to be significantly different.

## Results

### PCP tumors are in a state of high immune infiltration

**Figure 2:**
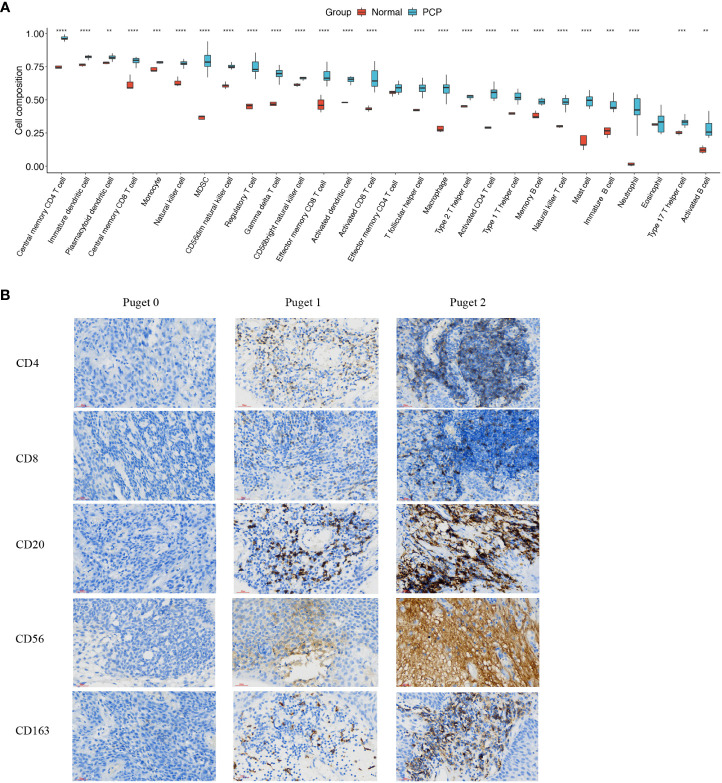
Infiltration of immune cells in PCP tumor. **(A)** Twenty-eight subtypes of tumor-infiltrating immune cells in PCP tumor samples compared with those in normal brain tissues. The following symbols indicate statistical significance: blank: *p* > 0.05; **p ≤ 0.01; ***p ≤ 0.001; ****p ≤ 0.0001. **(B)** Immune historical staining of 5 immune cell biomarkers in PCP samples of different Puget groups, 40×.

To assess immune infiltration level in PCPs, we performed bulk-RNA sequence in 11 PCP samples compared to normal brain tissues. Our results revealed that the infiltration of 28 subtypes of immune cells, except for effector memory CD4+ T cell and eosinophil, were significantly higher in PCP tumor tissues than in normal brain tissues (**Figure 2A**). To get more convincing evidence, after patients informed consent, we collected 8 PCP tumor samples for immune historical staining and 5 biomarkers of immune infiltration cells were detected including CD4, CD8, CD20, CD56, and CD163. As the results showed, these biomarkers were in a high expression level, which suggesting that there may be abundant immune cells infiltrated in PCPs (**Figure 2B**). These results proved that PCP tumors are in a state of high immune infiltration.

### Aggressive PCPs have particular immune microenvironment

**Figure 3:**
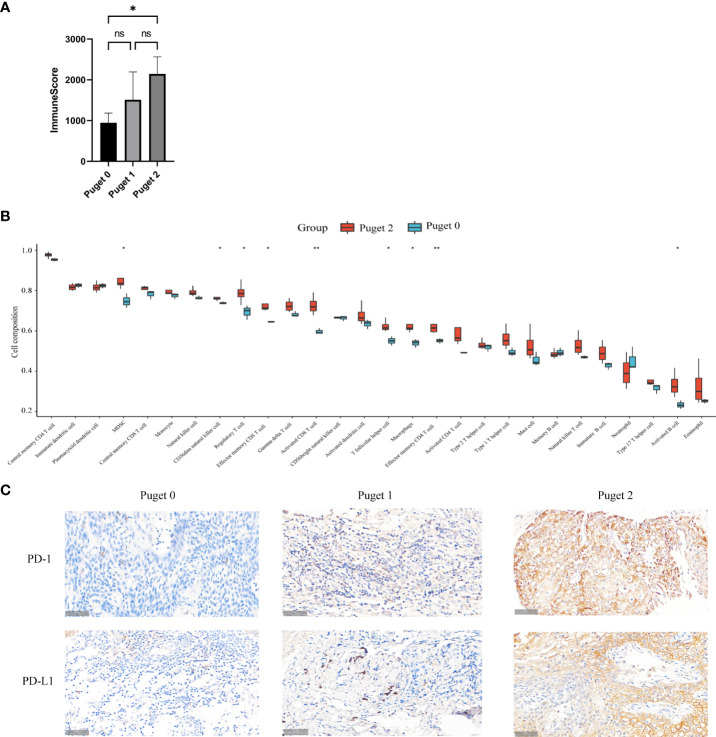
Aggressive PCPs have particular immune microenvironment. **(A)**. Three groups (Puget grade 0, n = 3; Puget grade 1, n = 4; Puget grade 2, n = 4) were analyzed using immune scores. ns: *p* > 0.05; *: *p* ≤ 0.05. **(B)** Twenty-eight subtypes of tumor-infiltrating immune cells in high aggressive groups (Puget grade 2, n = 4) compared with those in low aggressive groups (Puget grade 0, n = 3). The following symbols indicate statistical significance: blank: *p* > 0.05; *p ≤ 0.05; **p ≤ 0.01. **(C)**. IHC staining of PD-1 and PD-L1 in high aggressive PCP tissues compared to low aggressive PCP tissues, 40×.

To uncover the relevance of clinical features to immune infiltrates, we computed the immune scores of 11 PCP samples according to the R package ESTIMATE. PCP samples were divided into three groups according to Puget grading system (Puget grade 0, n = 3; Puget grade 1, n = 4; Puget grade 2, n = 4). Significant differences were found between low (Puget grade 0, n = 3) and high (Puget grade 2, n = 4) aggressive groups, with *p* = 0.0402 (**Figure 3A**). Furthermore, evaluation of the infiltration subtypes of immune cells between low and high aggressive groups showed that the extents of macrophages, MDSC, and activated CD8+ T, effector memory CD4+ and CD8+ T, CD56dim natural killer, regulatory T, T follicular helper, and activated B cells were significantly higher in high (Puget grade 2, n = 4) aggressive groups than in low (Puget grade 0, n = 3) aggressive groups (p < 0.05; **Figure 3B**). This implied there was a positive correlation between aggressiveness of PCP and tumor immune infiltration. The immunosuppressed function of neutrophil cells could be reasonable explanation to what we found. To investigate whether immune microenvironment in aggressive PCP is active, we detected the two most common immune checkpoint molecular PD-1 and PD-L1 with IHC staining. However, the expression levels of PD-1 and PD-L1 were high (**Figure 3C**). These results described a specific immune microenvironment of aggressive PCP tumors, in which there were plentiful immune cells infiltrated but low anti-tumor activity.

### Neutrophils infiltrated may lead to suppressive immune microenvironment in PCPs

**Figure 4:**
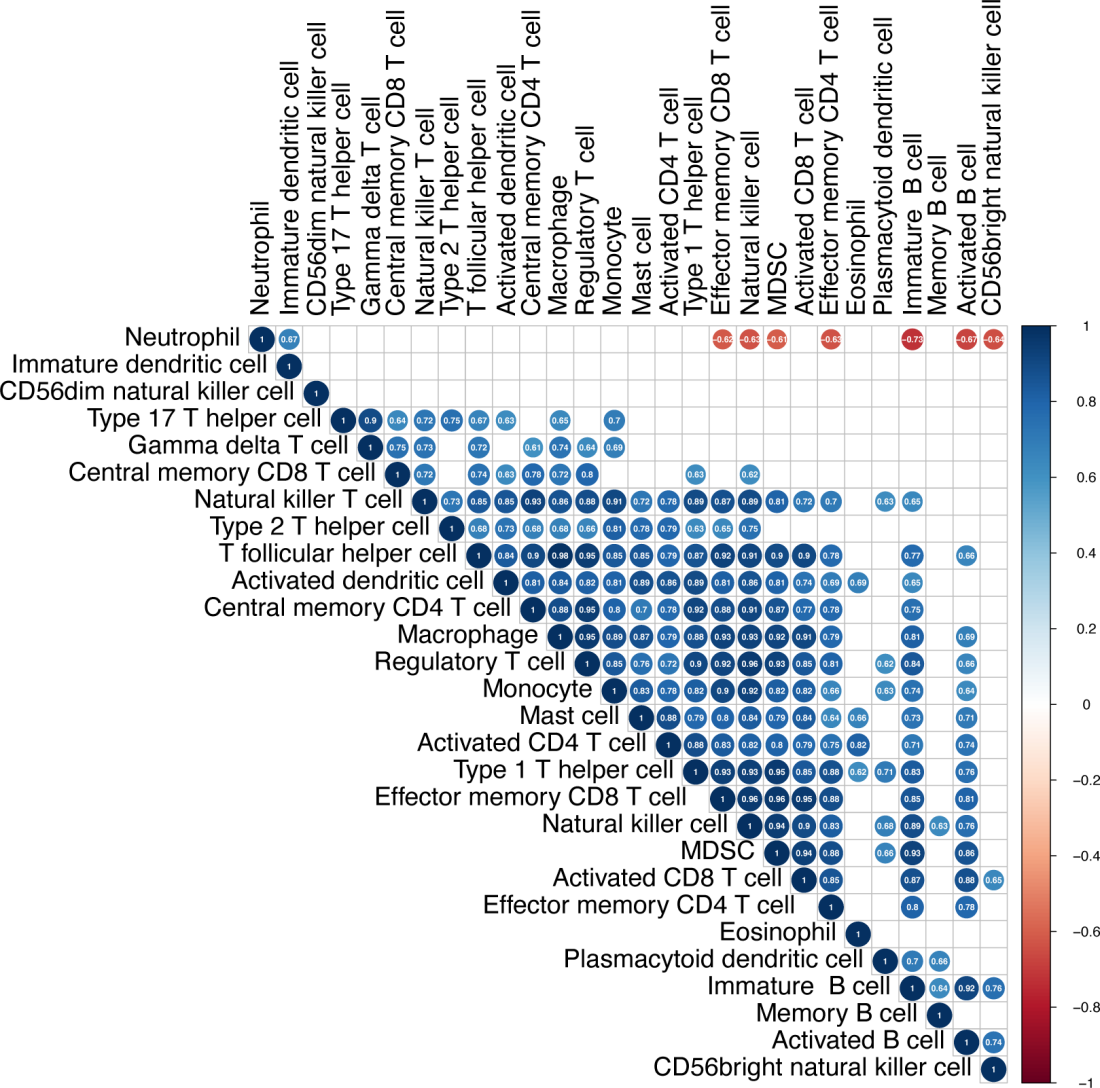
Infiltration of immune cells in PCP tumor. The heatmap shows correlations between the 28 immune cell subtypes. The strength of the correlation is indicated by a heatmap colored from red to blue. The numbers in each cell indicate the Pearson coefficients, while blank cells present the non-statistically significant numbers.

To get more detail about immune microenvironment of PCPs, we evaluated the possible correlation between the 28 immune cells in PCP samples. The heatmap showed that the infiltrate extents of the different tumor infiltration immune cell subgroups were moderate to strongly correlated. Remarkably, the extent of neutrophil cell infiltration was negatively correlated with some other immune cell groups (effector memory CD8+ T cell, natural killer cell, myeloid-derived suppressor cell (MDSC), effector memory CD4+ T cell, immature B cell, activated B cell, and CD56bright natural killer cell), (**Figure 4**). Based on this found, we bravely assumed that the suppressive immune microenvironment of PCPs could attributed to highly suppressive neutrophil infiltrated.

### IL-1A and IL-6 are associated with invasiveness of PCPs

**Figure 5:**
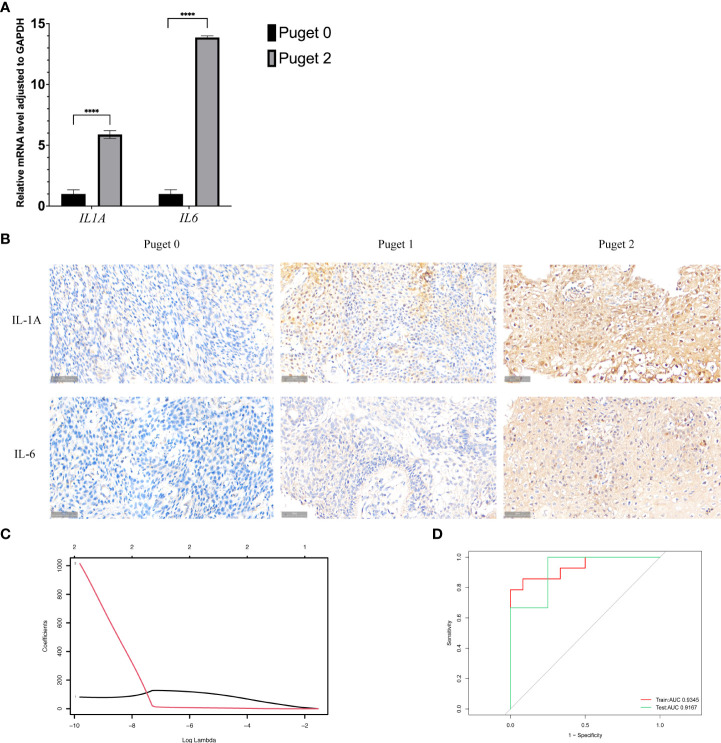
Interleukin-1α (*IL1A*) and interleukin-6 (*IL6*) in PCP tumors. **(A)**.Quantitative reverse transcriptase-polymerase chain reaction analysis was performed to analyze the mRNA expression levels of *IL1A* and *IL6* in Puget grade 0 group (n = 16) compared to Puget grade 2 group (n = 20), ****p ≤ 0.0001. **(B)**. IHC staining of IL-1A and IL-6 in PCP samples of different Puget groups, 40×. **(C)**. LASSO regression of *IL1A* and *IL6*. **(D)**. Receiver operating characteristic curve analysis shows the sensitivity and specificity for two inflammatory factors *IL1A* and *IL6*, as well as the area under the curve with 95% confidence intervals.

Based on what above-mentioned, we found that there were several types of immune cell highly infiltrated in aggressive PCPs. Most of them could produce inflammatory mediators IL-1A and IL-6, which can restrain immune reaction. We, therefore, hypothesized that the expression of these inflammatory cytokines was increased in PCP tumors, especially Puget grade 2. Then we performed qRT-PCR analysis to detect the transcription of *IL1A* and *IL6* in the low (Puget grade 0, n = 3) and high (Puget grade 2, n = 4) aggressive groups. Besides, the protein level of IL-1A and IL-6 in different aggressive PCP samples were detected by immunohistochemical staining. The results demonstrated that the mRNA levels and the protein levels of IL1A and IL6 were significantly increased (p<0.05) in the Puget grade 2 PCP group compared with the Puget grade 0 PCP group (**Figures 5A, B**).

Moreover, we brought *IL1A* and *IL6* into the LASSO regression model both in the training and testing sets. Through this model, we obtained the regression coefficient values of 50.96 and 221.78 for the inflammatory mediators *IL1A* and *IL6*, respectively (**Figures 5C**). A depiction of the LASSO scores for the training and testing datasets exhibited segregation of the low (Puget grade 0 group, n = 16) and high (Puget grade 2 group, n = 20) aggressive groups, and it demonstrated the good discriminating power of *IL1A* and *IL6*. Receiver operating characteristic (ROC) analysis was used to determine whether inflammatory mediators *IL1A* and *IL6* had any predictive value for distinguishing the invasiveness of tumors. The ROC curves of training and testing sets showed areas under the curve (AUC) values of 0.89 and 0.96 respectively, indicating the high accuracy (AUC > 0.9) of our models (**Figure 5D**).

### Identification of invasiveness-related genes

**Figure 6:**
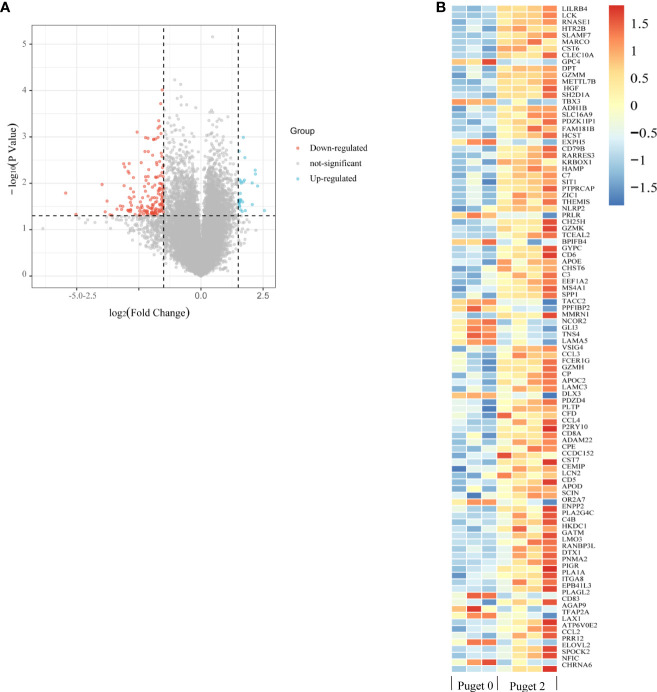
Enrichment analysis of differentially expressed genes (DEGs) related to invasiveness. **(A)** The volcano plot indicates DEGs between the low aggressive group (Puget grade 0, n = 3) and the high aggressive group (Puget grade 2, n = 4). Blue and red symbols classify the upregulated and downregulated genes, respectively, according to the criteria: |log2FC| > 1.5 and p-value < 0.05. **(B)** The heatmap summarizes the partly top differential gene expression in low and high aggressive groups.

We also analyzed the genes related to low and high aggressive groups and found 189 differentially expressed genes (DEGs), as shown by the volcano plot. Of these, 28 were upregulated and 161 were downregulated DEGs (**Figure 6A**). Heatmap shows some top differentially expressed genes in different PCP samples (**Figure 6B**).

### Enrichment analysis of invasiveness-related genes

**Figure 7:**
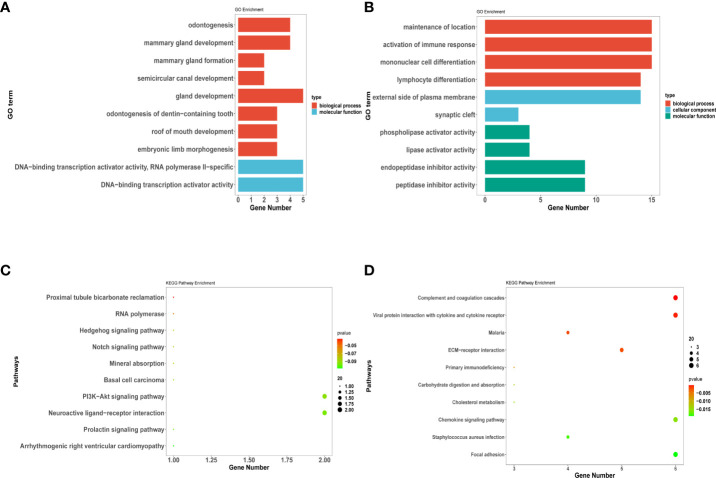
DEGs related to invasiveness analysis for Gene Ontology (GO) and Kyoto Encyclopedia of Genes and Genomes (KEGG) enrichment. **(A–D)** represent the enrichment analysis results of DEG genes involved in invasiveness. **(A) (C)** indicate the upregulated DEGs enriched in GO terms and KEGG pathway; **(B) (D)** indicate the downregulated DEGs enriched in GO terms and KEGG pathway. The main results of each term are shown, where the color indicates the significant degree of enrichment and the size indicates the number of genes enriched for each result.

To investigate the biological classifications of invasiveness-related genes, GO and KEGG enrichment analysis was performed by upregulated and downregulated genes with the R package “clusterprofile”. GO analysis results showed that changes in the BPs of upregulated genes were significantly enriched in odontogenesis, mammary gland formation, and mammary gland development. Moreover, changes in the MFs of upregulated genes were enriched only in the DNA-binding transcription activator and RNA polymerase II-specific and DNA-binding transcription activator activity. No enrichment was observed pertaining to changes in the CCs of upregulated genes (**Figure 7A**).

GO analysis results exhibited that changes in the BPs of downregulated genes were enriched in the maintenance of location and activation of the immune response. Moreover, changes in the CCs of downregulated genes were enriched in the external side of the plasma membrane and synaptic cleft. Additionally, changes in the MFs of downregulated genes were enriched only in the phospholipase and lipase activator activity (**Figure 7B**).

KEGG analysis revealed that the PI3K−Akt signaling and neuroactive ligand−receptor interaction pathways were significantly enriched in the upregulated genes (**Figure 7C**). Pathways such as viral protein interaction with cytokine and cytokine receptor, chemokine signaling pathway, and complement and coagulation cascades were significantly enriched in the downregulated genes (**Figure 7D**).

## Discussion

The tumor immune microenvironment was thought to play a crucial role in cancer development. A Stevens et al. firstly explored immune cells infiltrated in brain tumors including craniopharyngioma (12). Numerous studies have demonstrated a significant inflammatory response in ACP tumors, which contributes to adverse clinical outcomes, and confirmed the important role of inflammatory response in ACP tumor growth, invasion, and recidivism (14, 20, 28). Studies have shown that craniopharyngiomas are characterized by a high level of immunogenicity and infiltrated by several different immune cell types, which may be associated with the clinical outcome (29–33). Ros Whelan et al. have reviewed ever mounting evidence that inflammatory processes and the immune response play a critical role in the pathogenesis of both the solid and cystic portion of ACPs (34). But owing to a low incidence of PCPs compared with ACPs, there was not enough research on PCP tumor microenvironment and immune infiltration. Therefore, quantitative evaluation of tumor-infiltrating immunocytes may provide new opportunities for better stratification and prediction of prognosis in patients with PCP.

The aforementioned Puget grading system is essential for planning a surgical strategy for patients with PCP and avoiding irreversible hypothalamic damage. However, some PCPs that invaded into the hypothalamus have specific bionomics while Puget grading system only focus on magnetic resonance imaging. In most cases within our study, Puget 2 grade PCP tumors could always be characterized by inflammation signs such as tumor surrounded by irregular edema, increased tumor brittleness, and neutrophil infiltration, which were completely different from Puget 0 grade PCPs. Previous studies about ACP have suggested that the infiltration of immune cells could lead to carcinogenesis (35). Hence, we suspect that there may be a direct correlation between inflammatory infiltration and PCPs’ invasion. And our results showed that the immune score was improved in Puget 2 grade tumor group compared with Puget 0 tumor group (p < 0.05). So far, we have established a positive correlation between tumor invasion and inflammatory infiltration in PCP tumors.

By analyzing RNA sequencing data, the “ESTIMATE” algorithm was applied for assessing the infiltrating immune cells in PCP tumor samples for the first time, and we finally discovered that PCP tumors were in a state of high immune infiltration. Inflammatory cells such as lymphocytes, macrophages, and neutrophils were more abundantly present in the PCP tumor tissues than in the normal brain tissues. The IHC results proved these bioinformatic conclusions. Furthermore, we evaluated the possible correlation between the 28 immune cells. Remarkably, the extent of neutrophil cell infiltration was negatively correlated with some other immune cell groups. Reportedly, neutrophils are rare in ACP, whereas dense neutrophilic inflammation is present in 66% of PCP cases (27). Neutrophils can suppress antitumor immunity *via* the release of cytokines, and activated neutrophils may function as MDSCs in human tumors. Combined with the abovementioned tumor microenvironment results, we hypothesized that the suppressive immune microenvironment of PCPs could attributed to highly suppressive neutrophil infiltrated. However, the underlying mechanism remains unclear. Elucidating how neutrophil regulates other immune cells may be the key to better understanding the PCP tumor microenvironment.

Numerous cytokines, chemokines, and inflammatory mediators are expressed in human craniopharyngiomas in both the solid and cystic components of ACPs (14, 19, 36). According to some reports, elevated expression of the cytokines such as IL-1, IL-6, IL-8, IL-10, IL-18, TNF, and CXCL1 have been found in the cystic fluid of human ACPs (15, 16). By synthetically analyzing biological information obtained from the tumor samples, macrophages, MDSC, and activated CD8+ T, effector memory CD4+ and CD8+ T, CD56dim natural killer, regulatory T, T follicular helper, and activated B cells were significantly higher in high aggressive group (Puget grade 2, n = 4) compared to low aggressive group (Puget grade 0, n = 3), and most of these immune cells can generate IL-1α and IL-6 (37).We, therefore, hypothesized that the expression of these inflammatory cytokines was increased in PCP tumors, especially Puget grade 2. Therefore, we detected IL1A and IL6 gene expression by qRT-PCR and protein level by IHC. Compared with the Puget 0 grade tumor group, the expression of IL1A and IL6 in the Puget 2 tumor group was significantly increased. These results indicated that multiple components constitute the suppressive immune microenvironment around PCP cells, so that they can proliferate continuously. Targeting neutrophil or inflammatory cytokines IL-1A and IL-6 could be potential therapies to restore anti-tumor immune reaction in PCPs.

Meanwhile, IL-1A and IL-6 of Puget grade 2 tumor invasion to the hypothalamus were identified using LASSO regression. Here, the training, testing, and entire cohorts consisted of 10, 26, and 36 PCP tumor samples, respectively. Based on LASSO regression, a risk score model was designed to predict hypothalamic invasion in patients with PCP tumors. The predictive capability of *IL1A* and *IL6* was also successfully confirmed in the testing and training cohorts. ROC curve analysis showed that *IL1A* and *IL6* performed well in defining Puget grade 2 PCP tumors. These findings demonstrated that *IL1A* and *IL6* could provide robust diagnostic biomarkers to identify PCP patients with Puget 2 tumors.

In this study, the gene expression profiles were also integrated to identify the DEGs between Puget grade 0 and 2 PCP tumors using bioinformatics analysis. The enriched GO terms by DEGs analysis for BPs were maintenance of location, activation of the immune response, and odontogenesis. Coincidentally, John R. Apps et al. demonstrated the activation of inflammatory and odontogenic programs in ACP through tumor compartment transcriptomics (15). These results may prove the hypothesis raised by Qi Song-Tao that the process of craniopharyngioma calcification resembles that which occurs in osteogenesis/odontogenesis (38). The KEGG pathways enriched by DEGs analysis were the PI3K−Akt signaling pathway, chemokine signaling pathway, and cytokine−cytokine receptor interaction, which may have roles in the modulation of inflammatory infiltration in tumor tissue. However, further investigation is required to elucidate the specific mechanism by which infiltration leads to tumor invasion, which may also be relevant for further research and development of targeted drugs for PCPs.

Our study had some limitations. Owing to the small sample size of this experiment, which may cause bias error, our conclusion needs further confirmation with a large-sample investigation. Moreover, the analyzed transcriptome data were generated through bulk RNA sequencing, which leads to the loss of some information because of the heterogeneous nature of the tumor tissue. This may be explored in more detail with the aid of single-cell RNA-seq techniques.

In conclusion, to the best of our knowledge, this is the first study to describe the immune microenvironment of PCP tumors. Here, we determined that the PCP tumor was in a state of high immune infiltration but low action. Moreover, we found that neutrophils, IL-1A, and IL-6 constitute the suppressive immune microenvironment of PCPs. Besides, we established that the proposed IL1A and IL6 model is a robust tool for diagnosis prediction for PCP patients with Puget 2 tumors, which may help to define the degree of hypothalamic invasion.

## Data availability statement

The data presented in the study are deposited in the baidu netdisk (https://pan.baidu.com/s/19G7aaCizFIAcuvUdsUSoLQ), accession code: hxtp.

## Ethics statement

The studies involving human participants were reviewed and approved by The Ethics Committee of Beijing Tiantan Hospital of Capital Medical University. The patients/participants provided their written informed consent to participate in this study.

## Author contributions

YJ and LM contributed to data acquisition, analysis, figures presentation and drafting of the manuscript. YX, NQ and SR contributed to sample collection and data analysis. KC, BZ and WW participated in the process of data acquisition and qRT-PCR experiments. LC, HG and SG contributed to figures presentation, revision of the manuscript and the design of the study. All authors contributed to the article and approved the submitted version.

## Funding

This study is supported by the Beijing Municipal Science & Technology Commission (Grant No. Z191100006619087).

## Conflict of interest

The authors declare that the research was conducted in the absence of any commercial or financial relationships that could be construed as a potential conflict of interest.

## Publisher’s note

All claims expressed in this article are solely those of the authors and do not necessarily represent those of their affiliated organizations, or those of the publisher, the editors and the reviewers. Any product that may be evaluated in this article, or claim that may be made by its manufacturer, is not guaranteed or endorsed by the publisher.
